# Effect of periodontal therapy on lung function: a twelve-month follow-up intervention study

**DOI:** 10.1186/s12931-025-03246-1

**Published:** 2025-05-03

**Authors:** Anders Røsland, Randi J. Bertelsen, Joachim Heinrich, Stein Atle Lie, Andrei Malinovschi, Dagmar F. Bunæs

**Affiliations:** 1 Department of Clinical Dentistry, Section of Periodontics, University Of Bergen, Bergen, Norway; 2 Department of Clinical Dentistry, Centre for Translational Oral Research (TOR), University of Bergen, Bergen, Norway; 3 Department of Clinical Science, University Of Bergen, Bergen, Norway; 4 Oral health centre of expertise in Western Norway, Bergen, Norway; 5 Institute of Occupational, Social, and Environmental Medicine, University of Munich (LMU), Munich, Germany; 6 Department of Medical Sciences, Clinical Physiology, Uppsala University, Uppsala, Sweden

**Keywords:** Oral health, Periodontitis, Respiratory health, Small airway resistance, Lung function testing

## Abstract

**Background:**

Evidence suggest an inflammatory link between respiratory health and periodontitis. This study aims to evaluate the impact of periodontal therapy on lung function.

**Methods:**

Sixty-two never-smoking patients with mild periodontitis and without other medical conditions participated in this single-blind, prospective trial. Patients underwent periodontal therapy following an infection control approach. Lung function was measured using forced oscillation technique, assessing airway resistance and reactance, and spirometry evaluating FEV_1_, FVC, and FEV_1_/FVC. Lung function and fractional exhaled nitric oxide were assessed at baseline, three and six weeks, and every three months for a year. Periodontal parameters were recorded at baseline, six weeks, six and 12 months. Data were analysed using mixed-effects regression models.

**Results:**

Patients (mean age 36 years, 58% female) showed significant improvements in periodontal parameters (*p* < 0.001). Oscillometry revealed a significant decrease in airway resistance at 11 Hz and 19 Hz after six weeks, with further significant decreases throughout the study. Resistance at 5 Hz (R_5_) consistently declined, reaching significance at three months (*p* = 0.001). By one year, R_5_, R_11_, R_19_, and R_5 − 20_ showed significant reductions (all *p* < 0.05). Airway reactance at 5 Hz became less negative at three months (*p* = 0.002), while the reactance area (AX) decreased significantly at six months (*p* = 0.008). No significant changes were observed in spirometry or fractional exhaled nitric oxide.

**Conclusion:**

A decrease in airway resistance was observed after periodontal therapy, underscoring its positive impact on small airway function. These findings suggest that oral infection control is valuable for respiratory health in young adults before chronic conditions establish.

**Clinical trial registration:**

The trial was registered at ClinicalTrials.gov (NCT04781153) on February 19, 2021, prior to participant enrolment.

**Supplementary Information:**

The online version contains supplementary material available at 10.1186/s12931-025-03246-1.

## Background

Chronic respiratory diseases, leading causes of global morbidity and mortality [1], affect the airways and lungs. Periodontitis is a chronic, non-communicable disease that affects up to 50% of the global population [2]. It is initiated by accumulation and dysbiosis of dental biofilm, prompting an inflammatory host response that in turn leads to the destruction of tooth-supporting tissues [3].

Studies have shown associations between periodontitis and impaired lung health, including airflow obstruction [4], airflow limitation [5], and reduced spirometry indices like forced expiratory volume in the first second (FEV_1_) [6]. The suggested biological pathways are pathogens entering the lungs through micro-aspiration or systemic dissemination, triggering airway inflammation [7] leading to poorer respiratory health outcomes [8–10]. Additionally, shared risk factors may also play a role in linking periodontitis to respiratory health [11, 12]. Research, primarily in patients suffering from chronic obstructive pulmonary disease (COPD), indicates that dental treatment can improve lung function and reduce COPD exacerbations [13–16].

To our knowledge, no prior study has investigated the effect of periodontal therapy on lung function in a never-smoking population without overt chronic lung disease with focus on mild periodontitis. This is the most common form of periodontitis, affecting approximately 50% of the global population. Therefore, the primary aim of this study was to evaluate whether removing dental biofilm and achieving oral infection control in cases of mild periodontitis can lead to improved lung function.

## Methods

### Study design

The present study is a single-blind, prospective, longitudinal clinical trial designed to evaluate change in lung function following periodontal therapy. Sample size was calculated based on the primary outcome of forced expiratory volume in the first second (FEV_1_). Hypothesising that periodontal therapy would cause a minor change in FEV_1_, we designed the study to detect an absolute difference of 120 ml in FEV_1_. Assuming also that the standard deviation of the difference would be 150 ml, we calculated that a sample size of 52 was needed to have 80% power of observing a statistically significant result with a 5% level of significance. To account for 15% drop out within the population, we aimed to include 62 participants.

**Fig. 1:**
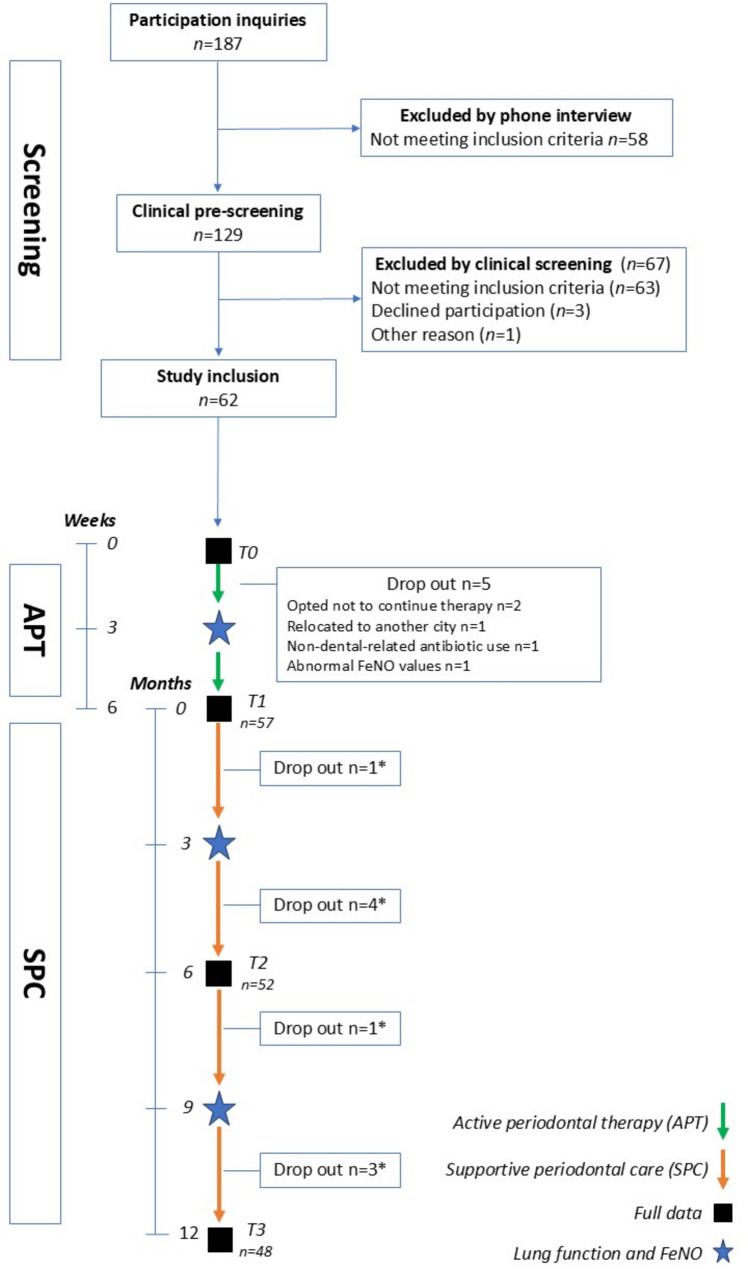
Flow chart of enrolment, periodontal therapy, data collection, and dropout timepoints and reasons for dropout

Flow-chart of enrolment, therapy, data collection and dropout reasons and timepoints are presented in Fig. 1. Following the active phase of periodontal therapy, patients were monitored at three-month intervals for one year. The trial was conducted in accordance with the Strengthening the Reporting of Observational Studies in Epidemiology (STROBE) guidelines to ensure comprehensive and transparent reporting. Ethical approval was obtained from the Regional Committee for Medical and Health Research Ethics in Western Norway (approval no. #94605) and the trial was registered at ClinicalTrials.gov (NCT04781153) on February 19, 2021, prior to inclusion. All participants provided written informed consent.

### Study participants

Recruitment of participants relied primarily on advertising through media channels, as well as through a review of eligible subjects from the university clinic’s patient database. A total of 187 individuals were contacted by phone for assessment of inclusion and exclusion criteria, 129 individuals underwent a clinical examination of which 62 fulfilled the inclusion criteria and consented to study inclusion. The examination and treatment were performed at the Department of Clinical Dentistry, University of Bergen between April 2021 to June 2024. Details on pre-study tests are described in the supplement.

Inclusion criteria: (1) Never-smoking individuals aged between 25 and 45 years; (2) good general systemic health defined as no history of systemic diseases or medications likely to interfere with study outcomes; (3) non-severe periodontitis (Stage I-II); (4) high dental plaque- and bleeding percentage (*≥* 50%).

Exclusion criteria were: (1) current or former smoking. Smoking history was assessed during the screening process, and participants who reported any current or former smoking during the interview were excluded; (2) current use of moist tobacco; (3) chronic lung diseases based on self-reported medical history. Chronic lung diseases were defined as physician-diagnosed, long-term respiratory conditions such as asthma, chronic obstructive pulmonary disease, or other persistent pulmonary disorders requiring ongoing treatment or follow-up; (4) symptoms of pollen allergy; (5) pregnancy; (6) systemic antibiotics last six months; (7) subgingival scaling last six months; (8) regularly use of oral antiseptic mouth rinse; (9) any current medical condition which may mediate the association between exposure and outcome.

Figure 1. Showing enrolment, periodontal therapy, data collection, and dropout timepoints during the whole trial. Full data: comprised a full-mouth periodontal charting, oral hygiene assessment, lung function testing and questionnaire. Lung function and FeNO: Forced oscillation technique (FOT), spirometry, and Fractional exhaled nitric oxide (FeNO) testing. Active periodontal therapy (APT): supra- and subgingival biofilm removal with a full-mouth disinfection protocol. Supportive periodontal care (SPC): proactive and preventive measure aimed at maintaining the health of the periodontium after active treatment and ensuring periodontal biofilm and inflammation control. T0: baseline, T1: six-week follow-up, T2: six-month follow-up, T3: twelve-month follow-up. * patient citing an inability to adhere to the follow-up regimen.

### Data collection

A full data collection comprised lung function measurements and assessment of levels of fraction of exhaled nitric oxide (FeNO), blood samples (serum), questionnaires, and a full-mouth periodontal charting. Lung function measurements, FeNO assessments, blood sample collection, and questionnaire completion were conducted on the same day, prior to the dental examination. The personnel assessing lung function were blinded to the participants’ periodontitis status. The first author (AR) collected the periodontal parameters blinded to lung function results. Participants were un-blinded to all variables.

Full data collection occurred at four time points: baseline (T0), six weeks (T1), six months (T2), and twelve months (T3) following the active periodontal therapy. During the follow-up time points SPC1 (supportive periodontal care 1, three months post-therapy) and SPC2 (supportive periodontal care 2, nine months post-therapy), patients underwent lung function measurements, oral hygiene assessment, and a full-mouth dental biofilm removal. At each clinical examination, patients were also asked about any changes in their medical history and medication status.

#### Lung function and FeNO measurements

Lung function, the primary outcome, and FeNO data were collected by trained fieldworkers at Research Unit for Health Surveys (RUHS), University of Bergen. FeNO measurements were performed according to standardized methods [17], prior to lung function assessment.

Respiratory impedance was measured by the forced oscillation technique (FOT) using Thorasys Tremoflo C-100 (Thorasys Medical Systems, Canada), prior to spirometry. During oscillometry testing, a stimulus is applied to the respiratory system at the mouth to measure the respiratory impedance in a passive manner [18]. Impedance is further split into its components of respiratory system resistance (Rrs) and reactance (Xrs). Rrs reflects airway diameter whereas Xrs reflects the elastic and inertive properties of the respiratory system [18]. Resistance from 5 to 20 Hz (R_5_, R_11_, R_19_, R_20_), the reactance at 5 Hz (X_5_), the resonant frequency (Fres) and the area under the reactance curve (AX) were calculated for each manoeuvre. To acquire the resistance of the distal airways, the difference between R_5_ and R_20_ was calculated. The coefficient of variation (CoV) for R_5_ was the main index of the reliability and repeatability of the impedance measurements, and measurements were conducted according to the 2020 European Respiratory Society guideline [18]. Further details can be found in the supplement.

Spirometry was performed in line with the American Thoracic Society/European Respiratory Society recommendations [19] using *ndd EasyOne Spirometer*. No bronchodilator medication was given to the subjects. To obtain optimal flow-volume curves the participants performed at least three, but no more than eight manoeuvres. The highest recorded values of forced expiratory volume in 1 s (FEV_1_), forced vital capacity (FVC) and the ratio of FEV_1_ and FVC (FEV_1_/FVC) were used in the analysis. Percent predicted values and z-scores were calculated from the 2012 Global Lung function Initiative (GLI) [20]. Based on the GLI 2012 recommendations for asymptomatic individuals, the lower limit of normal (LLN) was defined as the 2.5th percentile (z-score = -1.96). Normal lung function was defined as FEV_1_, FVC, and FEV₁/FVC z-scores all at or above the LLN. A restrictive spirometric pattern was defined as a preserved FEV_1_/FVC ratio (≥ LLN) with a reduced FVC (z-score < -1.96).

#### Periodontal variables

Dental and medical data (e.g. use of medications) were recorded during the periodontal examination by a single examiner (AR). The radiographic examination included intraoral radiographs with bitewing, and if needed supplementary periapical radiographs. The clinical examination comprised a full-mouth registration of probing pocket depth (PD) and clinical attachment loss (CAL) using a periodontal probe with 1 mm grading (Hu-Friedy PCPUNC157). PD was recorded at six sites per tooth as the distance in mm from the gingival margin to the base of the pockets, and CAL as the distance in mm from the cementum–enamel junction to the depth of the pocket (third molars excluded). Clinical periodontal inflammation and dental biofilm were assessed at four sites per tooth and expressed as the percentage of sites exhibiting bleeding on probing (BoP) [21] and visible supragingival dental plaque (PI) [22], using a disclosing agent (Curaprox, PCA 260). Periodontitis were diagnosed according to the 2017 World Workshop on the Classification of Periodontal and Peri-Implant Diseases and Conditions [23].

#### Other patient related assessments

Anthropometric data, serum samples, and questionnaire data were collected during the clinical examinations at RUHS. Further details can be found in the supplement. Serum high-sensitive C-reactive protein (CRP) levels (mg/L) were analysed using a high-sensitivity immunoturbidimetric assay (Cobas 8000, Roche Diagnostics) in the laboratory at Haukeland University Hospital, Bergen, Norway.

### Periodontal therapy

Periodontal therapy was conducted (by AR) in a stepwise manner according to the S3-level clinical practice guidelines from the European Federation of Periodontology [24]. Participants started Step 1 therapy immediately after baseline data collection (T0), which included oral hygiene instructions, removal of supragingival biofilm and calculus, and the elimination of local biofilm-retentive factors. To enhance treatment compliance, each patient was provided with a comprehensive oral hygiene kit for home use (details provided in the supplement). Step 2 periodontal therapy followed Step 1 and involved a modified full-mouth disinfection and scaling (FDIS) intervention approach [25]. Step 2 therapy commenced no later than three weeks following Step 1.

During Step 2 therapy, patients received a full-mouth supra- and subgingival biofilm removal using an ultrasonic scaler (SonicFlex, Piezolux/Sonosoft tip no.10, KaVo, USA) as well as hand instruments (Hu-Friedy, Chicago, IL, USA) within 24 h. Local anaesthesia (Xylocain Dental 2% adrenalin, Dentsply) was administrated, if needed. The intervention session was completed with a thorough cleansing with prophylactic paste (Prophy paste CCS RDA 170, Directa AB) and application of 1% chlorhexidine gel (Corsodyl, GlaxoSmithKline Consumer Healthcare, Denmark) in all sulci and pockets. The approximal sites were then flossed to carry the gel into the proximal contacts. At the end of the session, each patient was provided with a bottle of 0.2% chlorhexidine solution (Curaprox Perio Plus Forte CHX 0.2%) and a tube of 1% chlorhexidine gel (Corsodyl, GlaxoSmithKline Consumer Healthcare, Denmark) for home use. For a two-week period following therapy, participants were instructed to rinse with the chlorhexidine solution for one minute twice daily (morning and before bedtime) and to brush their tongues with chlorhexidine gel at bedtime. Between tooth brushing and chlorhexidine mouth wash, participants were instructed to rinse thoroughly with water for 30 s to avoid possible carryover interactions between toothpaste and chlorhexidine agents.

### Statistical analyses

For the analysis of the periodontal parameters, the patient was the unit of observation, meaning that BoP, PI, PD, and CAL was represented as the whole-mouth averages. All individuals had lung function data collected at multiple time-points. To assess changes in the repeated measurements over time, linear mixed-effects models were used, with individual as a random effect. The random mixed effects models also adjust for data missing at random (i.e. participants with loss to follow-up or drop-out).

Differences between groups over time were tested in the mixed effects model with the use of an interaction term between groups and time. P-values (two sided) less than 0.05 were considered statistically significant. All statistical analyses were conducted using Stata 17.0 (Stata Corporation, College Station, TX, USA).

## Results

The baseline characteristics of the study population are summarized in Table 1. A total of 62 participants were included, with a mean age of 35.8 years, and a predominance of female participants (58%). Fifty-six participants (90%) had normal lung function based on the LLN threshold, while one (1.6%) showed an obstructive spirometric pattern and three (4.8%) showed a restrictive pattern (data not shown). The mildest form of periodontitis (Stage I) was diagnosed in 21% of participants, while 79% were diagnosed with Stage II. When stratified by sex, statistically significant differences were observed in baseline FEV_1_ and FVC values, as well as in the absolute values of oscillometry variables R_5_, R_11_, and R_19_. Of the initial cohort, 48 patients (77.4%) successfully completed the trial (Fig. 1). An attrition analysis was conducted to compare baseline characteristics between subjects who dropped out and those who completed the trial (T0 to T3). No statistically significant differences were observed in lung function measurements between the two groups (Table S1). No adverse events were reported throughout the study.

**Table 1:** Baseline characteristics of study population stratified by sex

Baseline characteristics of study population		Sex		*p*-value
Variables	Total	Female	Male	
Participants	*n* = 62	36 (58%)	26 (42%)	
Mean age (SD)	35.8 (6.2)	36.1 (6.0)	35.3 (6.5)	0.64
Married or cohabitant	38 (63.3%)	22 (57.9%)	16 (42.1%)	0.66
Education at University Level	51 (85.0%)	32 (62.8%)	19 (37.2%)	0.56
Employed	50 (83.3%)	30 (40%)	20 (60%)	0.73
Mean body mass index in kg/m^2^ (SD)	27.4 (5.1)	27.5 (5.3)	27.3 (5.1)	0.89
Mean height in cm (SD)	172 (9.9)	165.6 (6.3)	181.2 (5.9)	**< 0.001**
Mean weight in kg (SD)	81.5 (18.7)	75.7 (17.3)	89.6 (17.7)	**0.003**
**Severity of periodontitis**				0.77
Stage I	13 (21.0%)	8 (61.5%)	5 (38.5%)	
Stage II	49 (79.0%)	28 (57.1%)	21 (42.9%)	
**Exercise**				0.38
Once a week or less 2–3 times per week Almost every day	19 (32.2%) 25 (42.4%) 15 (25.4%)	9 (47.4%) 17 (68%) 9 (60%)	10 (52.6%) 8 (32%) 6 (40%)	
**Absolute values of spirometry variables**				
Mean FEV_1_ (SD) Mean FVC (SD) Mean FEV_1_/FVC (SD)	3.55 (0.77) 4.53 (1.02) 0.79 (0.06)	3.1 (0.44) 3.9 (0.60) 0.79 (0.06)	4.2 (0.62) 5.4 (0.90) 0.78 (0.05)	**< 0.001** **< 0.001** 0.58
**%predicted spirometry variables**				
Mean %predicted FEV_1_ (SD) Mean %predicted FVC (SD)	94.3 (10.8) 98.6 (11.4)	95.5 (10.8) 100.2 (10.7)	92.6 (10.9) 96.4 (12.1)	0.30 0.18
**Absolute values of oscillometry variables**				
Mean R_5_ (SD) Mean R_11_ (SD) Mean R_19_ (SD)	3.22 (1.10) 3.00 (0.84) 2.78 (0.72)	3.53 (1.12) 3.30 (0.85) 3.10 (0.70)	2.80 (0.90) 2.60 (0.70) 2.40 (0.52)	**0.006** **0.001** **< 0.001**
**%predicted oscillometry variables**				
Mean R_5_%predicted (SD) Mean R_11_%predicted (SD) Mean R_19_%predicted (SD)	102.85 (26.44) 102.37 (23.47) 93.10 (19.40)	103.2 (29.2) 102.9 (25.8) 94.3 (20.7)	102.4 (22.3) 101.6 (19.9) 91.4 (17.5)	0.91 0.84 0.58
**Fraction of Exhaled Nitric Oxide (FeNO)**				
Mean FeNO in parts per billion (SD)	19.01 (14.35)	18.2 (16.5)	20.1 (10.9)	0.61
**Periodontal variables**				
Mean BoP in % (SD) Mean PI in % (SD) Mean PD in mm (SD) Mean CAL in mm (SD)	63.01 (1.62) 62.0 (1.90) 2.48 (0.02) 2.70 (0.03)	62.8 (14.1) 61.8 (10.4) 2.47 (0.20) 2.68 (0.20)	63.5 (13.7) 62.4 (10.2) 2.48 (0.18) 2.73 (0.18)	0.84 0.82 0.86 0.39
Mean high-sensitive C-reactive protein (mg/L)	1.70 (2.34)	1.67 (1.96)	1.71 (2.87)	0.94

Table 1. Data are presented as *n* (%) or mean and standard deviations (SD). FEV_1_/FVC is unitless and has the formula FEV_1_(L)/FVC (L). Percent predicted values were calculated from the 2012 Global Lung function Initiative. P-values are calculated by t-test (continuous variables) and Chi-square tests (categorical variables). Abbreviations: FEV_1_: forced expiratory volume in the first second, FVC: forced vital capacity, R_5_: airway resistance measured at 5 Hz, R_11_: airway resistance measured at 11 Hz, R_19_: airway resistance measured at 19 Hz. BoP: bleeding on probing, PI: plaque index, PD: periodontal pocket depth, CAL: clinical attachment loss.

### Periodontal parameters

Periodontal therapy significantly reduced the mean values of BoP, PI, PD and CAL (all *p* < 0.001) throughout the trial, compared to baseline measurements (Table 2). No statistically significant differences in the periodontal parameters were observed between sex (Figure S1).

**Table 2:** Periodontal variables from baseline (T0) to twelve-month follow-up (T3)

	T0	T1	*p*-value	T2	*p*-value	T3	*p*-value
	*n* = 62	*n* = 57		*n* = 52		*n* = 48	
**Periodontal variables**							
BoP in % (SE)	63.10 (1.62)	25.00 (1.68)	< 0.001	28.55 (1.73)	< 0.001	30.01 (1.78)	< 0.001
PI in % (SE)	62.00 (1.90)	22.15 (1.96)	< 0.001	23.06 (2.04)	< 0.001	27.30 (2.11)	< 0.001
PD in mm (SE)	2.48 (0.02)	2.14 (0.02)	< 0.001	2.15 (0.02)	< 0.001	2.16 (0.02)	< 0.001
CAL in mm (SE)	2.70 (0.03)	2.56 (0.03)	< 0.001	2.53 (0.03)	< 0.001	2.44 (0.03)	< 0.001

Table 2. Periodontal variables from baseline (T0) to twelve-month follow-up (T3). Data are presented as mean and standard error (SE). Abbreviations; BoP: bleeding on probing, PI: plaque index, PD: periodontal pocket depth, CAL: clinical attachment loss. Results from a mixed-effects model indicate the statistical significance of differences in means between timepoints, with baseline (T0) as the reference.

### Lung function and FeNO measurements following periodontal therapy

**Fig. 2:**
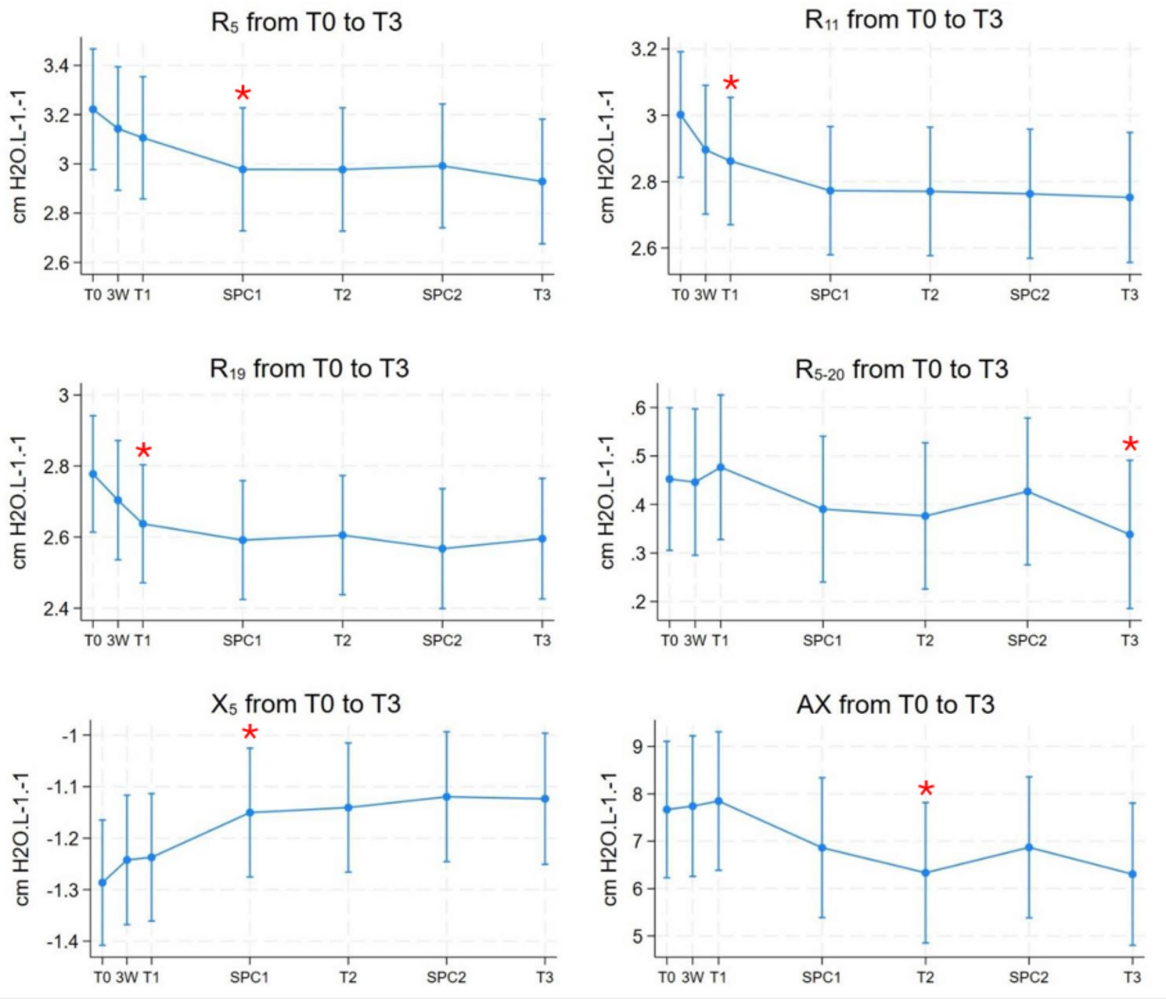
Graphs showing change in oscillometry variables from baseline (T0) to T3

**Fig. 3:**
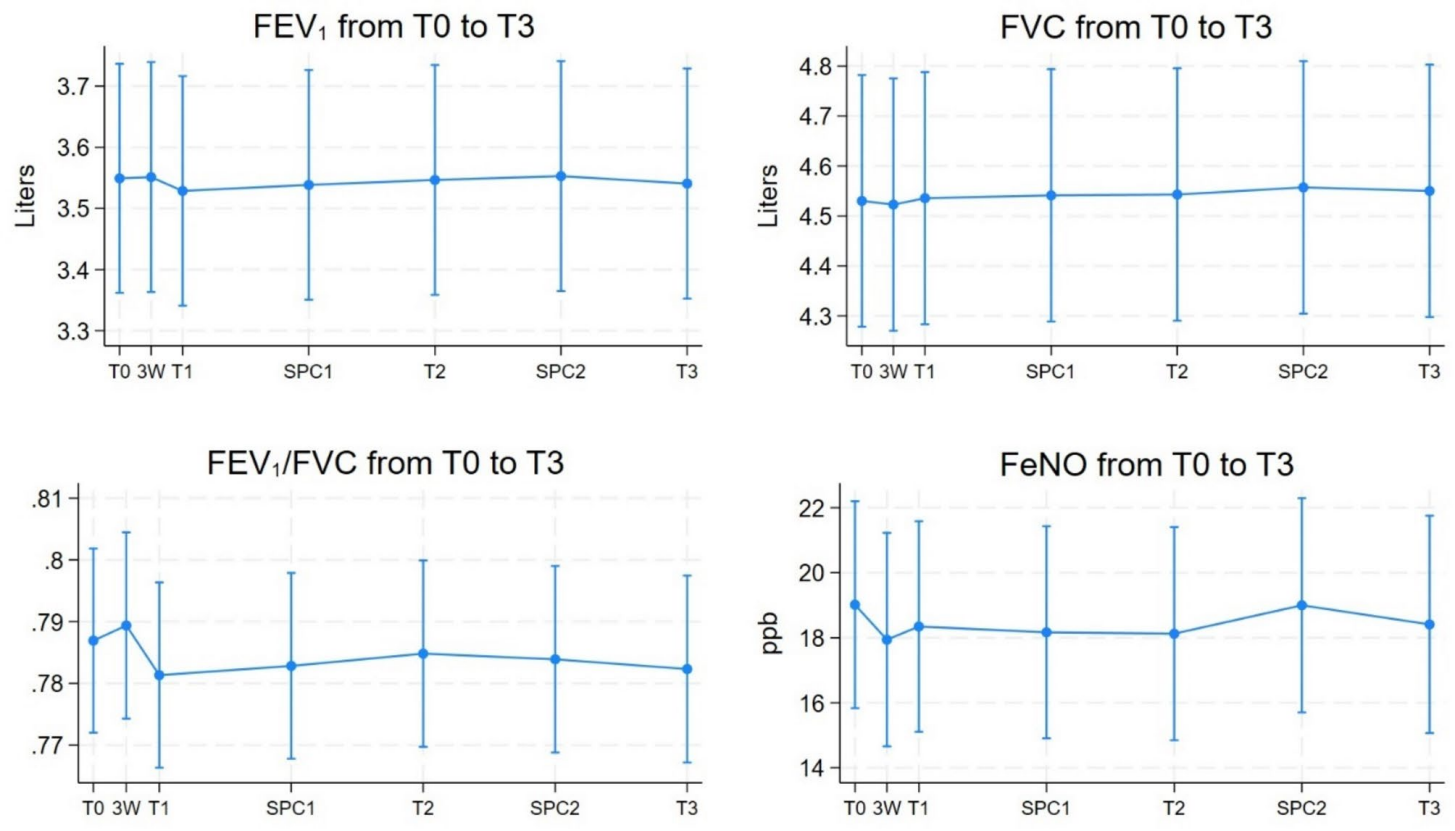
Graphs showing change in FEV_1_, FVC, FEV_1_/FVC and levels of FeNO from baseline (T0) to T3

The mean absolute values for lung function and FeNO measurements from baseline (T0) to twelve-month follow-up (T3) are presented in Table 3. Oscillometry testing revealed a decrease in airway resistance across all frequencies (Fig. 2). A statistically significant reduction in resistance was noted at 11 Hz and 19 Hz after six weeks (T1), with further decreases throughout the trial. Airway resistance at 5 Hz (R_5_) showed a consistent decline at all timepoints, reaching statistical significance three months after T1 (SPC1). One year following therapy, measurements of R_5_, R_11_, and R_19_ decreased by 9.3%, 8.3%, and 6.8%, respectively (all *p* < 0.05). Additionally, there was a statistically significant reduction in R_5 − 20_ at T3 (*p* = 0.02). Airway reactance at 5 Hz (X_5_) showed improvements (less negative values) at all timepoints compared to baseline, reaching statistical significance at SPC1. Correspondingly, the reactance area (AX) showed an overall reduction from T0 to T3, with a statistically significant decrease six months after therapy (T2) (Fig. 2). No statistically significant changes were observed in FEV_1_, FVC, FEV_1_/FVC, or FeNO levels following periodontal therapy (*p* > 0.05) (Fig. 3).

Figure 2. Graphs showing marginal means with 95% confidence intervals. Abbreviations: R_5_: airway resistance measured at 5 Hz, R_11_: airway resistance measured at 11 Hz, R_19_: airway resistance measured at 19 Hz, R_5 − 20_: the difference in airway resistance measured at 20 Hz and 5 Hz, X_5_: reactance measured at 5 Hz, AX: reactance area. T0: baseline, 3 W: three-week control, T1: follow-up at six weeks, SPC1: supportive periodontal care 1, conducted three months after therapy, T2: six months follow-up, SPC2: supportive periodontal care 2, conducted nine months after therapy, T3: twelve-month follow-up. * indicating *p* < 0.05.

Figure 3. Graphs showing marginal means with 95% confidence intervals. Abbreviations: FEV_1_: forced expiratory volume in the first second, FVC: forced vital capacity. FEV_1_/FVC is unitless and has the formula FEV_1_(L)/FVC (L), FeNO: fraction of exhaled nitric oxide, T0: baseline, 3 W: three-week control, T1: follow-up at six weeks, SPC1: supportive periodontal care 1, conducted three months after therapy, T2: six months follow-up, SPC2: supportive periodontal care 2, conducted nine months after therapy, T3: twelve-month follow-up.

**Table 3:** Absolute lung function measurements by spirometry and forced oscillation technique from baseline (T0) to twelve-month follow-up (T3)

Variables	T0	T1	*p*-value	SPC1	*p*-value	T2	*p*-value	SPC2	*p*-value	T3	*p*-value
	*n* = 62	*n* = 57		*n* = 55		*n* = 52		*n* = 50		*n* = 48	
**Spirometry**											
FEV_1_ in L (SE)	3.55 (0.09)	3.53 (0.09)	0.35	3.54 (0.09)	0.62	3.55 (0.09)	0.92	3.55 (0.09)	0.86	3.54 (0.09)	0.74
FVC in L (SE)	4.53 (0.13)	4.53 (0.13)	0.86	4.54 (0.13)	0.68	4.54 (0.13)	0.63	4.55 (0.13)	0.29	4.55 (0.13)	0.43
FEV_1_/FVC (SE)	0.786 (0.008)	0.781 (0.008)	0.07	0.783 (0.008)	0.2	0.785 (0.008)	0.55	0.784 (0.008)	0.37	0.782 (0.008)	0.16
**Oscillometry**											
R_5_ (SE)	3.22 (0.13)	3.10 (0.13)	0.1	2.97 (0.13)	**0.001**	2.98 (0.13)	**0.001**	3.00 (0.13)	**0.002**	2.92 (0.13)	**< 0.001**
R_11_ (SE)	3.00 (0.10)	2.86 (0.10)	**0.01**	2.77 (0.10)	**< 0.001**	2.77 (0.10)	**< 0.001**	2.77 (0.10)	**< 0.001**	2.75 (0.10)	**< 0.001**
R_19_ (SE)	2.78 (0.08)	2.63 (0.08)	**0.005**	2.59 (0.08)	**< 0.001**	2.60 (0.08)	**0.001**	2.57 (0.08)	**< 0.001**	2.59 (0.08)	**0.001**
R_5_-R_20_ (SE)	0.45 (0.07)	0.47 (0.08)	0.63	0.39 (0.08)	0.19	0.37 (0.08)	0.11	0.43 (0.08)	0.6	0.33 (0.08)	**0.02**
F_res_ (SE)	16.73 (0.60)	16.90 (0.61)	0.72	16.31 (0.62)	0.3	16.04 (0.62)	0.09	16.57 (0.62)	0.69	16.18 (0.63)	0.19
X_5_ (SE)	-1.29 (0.06)	-1.24 (0.06)	0.26	-1.15 (0.06)	**0.002**	-1.14 (0.06)	**0.001**	-1.12 (0.06)	**< 0.001**	-1.12 (0.06)	**< 0.001**
AX (SE)	7.66 (0.73)	7.84 (0.75)	0.71	6.86 (0.75)	0.1	6.33 (0.76)	**0.008**	6.87 (0.76)	0.11	6.30 (0.77)	**0.008**
FeNO (SE)	19.01 (1.62)	18.34 (1.62)	0.525	18.16 (1.65)	0.424	18.12 (1.67)	0.413	19.00 (1.68)	0.986	18.41 (1.70)	0.602

Table 3. Absolute measurements by spirometry, forced oscillation technique and FeNO from baseline (T0) to T3. Results from mixed-effects model presented as mean and standard error (SE). P-values from mixed-effects model indicate the statistical significance of differences in means between timepoints, with baseline (T0) as the reference. Abbreviations: FEV_1_: forced expiratory volume in the first second, FVC: forced vital capacity, R_5_: airway resistance measured at 5 Hz, R_11_: airway resistance measured at 11 Hz, R_19_: airway resistance measured at 19 Hz, R_5_-R_20_: the difference in airway resistance measured at 20 Hz and 5 Hz, F_res_: resonance frequency measured in Hz, X_5_: reactance measured at 5 Hz, AX: reactance area. FEV_1_/FVC is unitless and has the formula FEV_1_(L)/FVC (L). T0: baseline, T1: follow-up at six weeks, SPC1: supportive periodontal care 1 conducted three months after therapy, T2: six months follow-up, SPC2: supportive periodontal care conducted nine months after therapy, T3: twelve-month follow-up. FeNO: fraction of exhaled nitric oxide has the unit parts per billion. Variables R_5_, R_11_, R_19_, R_5_-R_20_, X_5_, and AX have the unit cm H2O.L^− 1^.s^− 1^.

### Additional analyses of lung function outcomes and C-reactive protein

Out of the 373 R_5_-observations collected across all participants and time points (baseline to T3), 98.7% exhibited a coefficient of variation (CoV) below 15%. At baseline, one participant had an R_5_ CoV exceeding 15%. Participants were stratified into two groups based on their baseline CoV: those with a CoV *≤* 10% and those with a CoV > 10%. An interaction analysis was performed to assess the treatment effect between these groups. The interaction term was not statistically significant for either resistance or reactance measurements (Figure S2). We stratified participants based on periodontal diagnosis (stage I or II) and evaluated potential differences in oscillometry and spirometry outcomes (Figure S3). The interaction analysis showed no statistically significant differences in either the oscillometry or spirometry variables between the strata (*p* > 0.05).

No significant reduction in CRP levels was observed at T1, and no further significant changes were detected throughout the trial period (*p* > 0.05) (Figure S4). However, when participants were stratified by baseline CRP levels above or below the median (0.85 mg/L), a statistically significant difference in treatment effects was observed for R_5_, X_5_, and AX (all *p* < 0.05) between these strata. No significant effect was found for R_11_ or R_19_ (all *p* > 0.05) (Figure S5).

## Discussion

In this novel study addressing the effect of periodontal biofilm control on lung function in healthy individuals, we could find an improvement in respiratory resistance and reactance, but no significant differences in spirometry. Improvements in respiratory resistance were evident as early as six weeks after treatment, with continued enhancement throughout the one-year follow-up period. The observed reduction in resistance, despite the absence of significant changes in spirometry, may be explained by the higher sensitivity of oscillometry in detecting early or subtle changes in lung function.

Multiple studies in general populations have shown an association between periodontitis and lower levels of FEV_1_ [6], increased risk of airflow obstruction [4], and reduced lung volumes and airflow limitation [5]. A recent systematic review by Molina et al. found positive associations between periodontitis and several lung disorders [26]. In addition to the epidemiological evidence, a small number of intervention studies have shown beneficial effects of periodontal interventions on individuals suffering from COPD [13–15]. Even though those intervention studies have been on COPD, the findings suggest that oral health is closely correlated with key indicators of lung disease, including exacerbations and lung function.

Studies have demonstrated that oscillometry is more effective in detecting early or subtle changes in lung function, particularly in small airways, as seen in conditions like asthma and COPD [27]. Oscillometry parameters may detect airflow obstruction in the peripheral airways before changes become evident in traditional spirometry, making it a more sensitive tool for capturing early changes [28]. The Rrs-parameters measure respiratory resistance at different frequencies, with R_5_ reflecting both central and peripheral airway resistance, and R_19_ primarily assessing central airway resistance. As such, FOT may be better suited for identifying gradual improvements in smaller airways. Additionally, the interaction analysis revealed that individuals with higher baseline airway resistance benefited more from periodontal treatment. This finding underscores the potential importance of integrating periodontal treatment into the management of patients with pre-existing airway obstructions, or those at risk.

Periodontitis, characterized by oral microbiota dysbiosis, has been linked to chronic respiratory diseases due to the close anatomical proximity of the oral and respiratory tracts and the risk of microaspiration of pathogens [29]. The most common route by which the oral cavity may influence pulmonary function is the aspiration of saliva containing oral bacteria into the lung [7, 30]. In addition to direct aspiration, pulmonary pathogens can colonize the dental biofilm supporting the idea that the oral cavity may act as a reservoir for pathogens associated with respiratory diseases, particularly in high-risk patients [31]. Elevated systemic host inflammatory response are linked to both periodontitis [10] and reduced lung function [9]. Moreover, released inflammatory cytokines from periodontal tissues may alter the respiratory epithelium, promoting respiratory pathogen growth and increasing susceptibility to lower airway infections [32]. Oxidative stress, a common feature of both periodontitis and lung diseases such as COPD, may play a role in the shared pathophysiology of these conditions. It contributes to lung tissue damage, impairs repair mechanisms, and exacerbates respiratory dysfunction, further linking oral and respiratory health [33].

Periodontal treatment has been shown to reduce systemic inflammation in otherwise systemically healthy patients [34]. In our study, we observed an overall non-significant decrease in CRP levels from baseline to the one-year follow-up (T3). This contrasts with previous studies that reported significant reductions in CRP following periodontal therapy, likely due to differences in study populations; prior studies included individuals with more severe periodontitis and higher baseline CRP levels than our cohort [34, 35]. Notably, our data indicate that patients with baseline CRP levels above the median experienced more pronounced benefits from treatment, with significant improvements in R_5_, X_5_, and AX measurements compared to patients with a CRP below median. By enhancing periodontal health and reducing systemic inflammation, it is plausible that inflammatory processes in the respiratory system may also be mitigated.

Chlorhexidine (CHX), a common antimicrobial in dental care, has demonstrated clinical benefits in one-stage full-mouth disinfection compared to traditional tooth cleaning methods [36]. The use of CHX targets not only periodontal pockets but also other bacterial reservoirs, such as the tongue and oral mucosa, thereby reducing the risk of recontamination in treated areas. In this study, periodontal treatment positively impacted periodontal health, consistent with previous findings on non-surgical therapy using the FDIS approach [37]. However, concerns exist about CHX’s potential to disrupt the oral microbiome by reducing beneficial bacteria and promoting less favourable ones [38], which may create a more acidic and harmful oral environment [39], along with possible systemic effects [40]. Despite this, adherence to the treatment protocol was excellent, and the risk of CHX-related adverse effects was considered very low.

### Strengths and limitations

The study possesses several strengths. Its longitudinal investigator-masked design, coupled with close follow-up throughout the intervention period, enables the comprehensive evaluation of the airway resistance before, during, and after the intervention. By targeting a population without lung disease but mild forms of periodontitis, elevated gingival inflammation, and high levels of dental biofilm, the study broadens the relevance of its findings. This focus enhances the generalizability of the results, as these periodontal conditions are widespread affecting many who do not have underlying respiratory disease. The positive outcomes observed in the present study could indicate benefits with wide-ranging implications for public health, supporting the role of periodontal care as a potential preventive measure for respiratory health across the general population.

Further strengths of this study include the use of FOT-measurements, which provide additional insight into airway function. Respiratory oscillometry measures identify functional abnormalities in symptomatic subjects despite normal airflow by spirometry and have been shown to correlate with clinical symptoms [41]. Moreover, by including healthy, never-smoking individuals aged 25–45, who have reached maximum lung capacity [42], minimizing potential confounding effects of age-related lung function decline and factors such as smoking which is a shared risk factor for both periodontitis and respiratory disease [43]. There were no observed differences in the participants’ physical exercise frequency or duration throughout the trial, suggesting that any potential effects on lung health outcomes remained consistent during the intervention period (data not shown). None of the patients reported using medications, such as inhalers or bronchodilators, during the follow-up period.

The study has several limitations that warrant consideration. First, the lack of a control group complicates the attribution of improvements in airway resistance solely to the intervention. All patients received periodontal therapy, and no untreated control group was included. This decision was grounded in ethical considerations regarding the progressive nature of periodontitis. Withholding treatment would have posed significant ethical concerns, particularly as standard periodontal therapies are well-established in clinical practice and withholding treatment could potentially harm participants. Instead, a pre-post study design was employed, where participants acted as their own controls, and baseline measures were used to evaluate the effects of the therapy on lung function. This approach allowed us to balance the ethical obligation to provide necessary care with the scientific aim of exploring the link between periodontal therapy and respiratory outcomes.

Another limitation of the study is the lack of established thresholds for minimal clinically important difference (MCID) for oscillometry parameters in healthy individuals. Although our findings of significant improvements in Rrs and Xrs over 12 months following periodontal therapy fall below the MCID thresholds for asthmatic patients [44], it is important to emphasize that our study focused on systemically healthy individuals without overt respiratory diseases. Nevertheless, the observed improvements, though modest compared to thresholds for diseased populations, may still indicate meaningful physiological benefits, especially if the trends continue beyond 12 months.

Unrecorded external factors may have contributed to the observed changes, complicating the interpretation of results. Changes in lifestyle behaviours such as diet, which were not part of the data collection, could introduce confounding effects. Additionally, selection bias may be present as participants who volunteered may systematically differ from non-participants, potentially affecting generalizability. Moreover, the onset of the COVID-19 pandemic presented logistical challenges, including appointment rescheduling due to only mild respiratory symptoms among participants. On the other hand, strict adherence to infection control measures ensured that respiratory infections did not impact lung function assessments during the study.

Within the limitations of the present study, our longitudinal investigator-masked clinical trial demonstrated a statistically significant decrease in airway resistance following periodontal therapy. These findings underscore the potential importance of periodontal biofilm control in managing respiratory health. However, the absence of a control group limits our ability to establish causal relationships between periodontal therapy and respiratory health outcomes. Therefore, caution is warranted in interpreting these findings as generalizable to broader populations. Future large-scale randomized controlled trials, incorporating both short-term and long-term follow-up periods, are warranted to further elucidate the impact of periodontitis prevention and treatment on respiratory outcomes.

## Electronic supplementary material

Below is the link to the electronic supplementary material.

Supplementary Material 1

## Data Availability

Due to Norwegian ethical and legal restrictions, all the data underlying the findings in our study cannot be made publicly available. Request for data access can be directed to the corresponding author.

## Abbreviations

AX: Reactance area
BoP: Bleeding on probing
CAL: Clinical attachment loss
CHX: Chlorhexidine
CoV: Coefficient of variance
CRP: High-sensitive C-reactive protein
COPD: Chronic obstructive pulmonary disease
FDIS: Full-mouth disinfection and scaling
FeNO: Fraction of exhaled nitric oxide
FEV_1_: Forced expiratory volume in the first second
FOT: Forced oscillation technique
Fres: The resonant frequency
FVC: Forced vital capacity
MCID: Minimal clinically important difference
PD: Probing pocket depth
PI: Plaque index
Rrs: Respiratory system resistance
RUHS: Research Unit for Health Surveys
Xrs: Respiratory system reactance
R_5_: The resistance measured at 5 Hz
R_11_: The resistance measured at 11 Hz
R_19_: The resistance measured at 19 Hz
R_20_: The resistance measured at 20 Hz
SPC: 1 supportive periodontal care 1, three months post-therapy
SPC2: Supportive periodontal care 2, nine months post-therapy
STROBE: Strengthening the Reporting of Observational Studies in Epidemiology
X_5_: The reactance measured at 5 Hz

## References

[1] Soriano JB, Kendrick PJ, Paulson KR, et al. Prevalence and attributable health burden of chronic respiratory diseases, 1990–2017: a systematic analysis for the global burden of disease study 2017. Lancet Respiratory Med. 2020;8(6):585–96. 10.1016/S2213-2600(20)30105-3 PMC7284317 32526187

[2] Dumitrescu AL, Editorial. Periodontal Disease - A public health problem. Front Public Health. 2015;3:278. 26779473 10.3389/fpubh.2015.00278 PMC4705816

[3] Papapanou PN, Sanz M, Buduneli N, et al. Periodontitis: consensus report of workgroup 2 of the 2017 world workshop on the classification of periodontal and Peri-Implant diseases and conditions. J Clin Periodontol. 2018;45(Suppl 20):S162–70. 29926490 10.1111/jcpe.12946

[4] Røsland A, Bertelsen RJ, Bunaes DF et al. Periodontitis is associated with airflow obstruction in the Malmö offspring dental study. J Clin Periodontol. 2023. 10.1111/jcpe.13886 37837290

[5] Holtfreter B, Richter S, Kocher T, et al. Periodontitis is related to lung volumes and airflow limitation: a cross-sectional study. Eur Respir J. 2013;42(6):1524–35. 23222882 10.1183/09031936.00109112

[6] Lee WC, Fu E, Li CH, et al. Association between periodontitis and pulmonary function based on the third National health and nutrition examination survey (NHANES III). J Clin Periodontol. 2020;47(7):788–95. 32390194 10.1111/jcpe.13303

[7] Imai K, Iinuma T, Sato S. Relationship between the oral cavity and respiratory diseases: aspiration of oral bacteria possibly contributes to the progression of lower airway inflammation. Japanese Dent Sci Rev. 2021;57:224–30. 10.1016/j.jdsr.2021.10.003 PMC8566873 34760030

[8] Gläser S, Ittermann T, Koch B, et al. Airflow limitation, lung volumes and systemic inflammation in a general population. Eur Respir J. 2012;39(1):29–37. 21719491 10.1183/09031936.00009811

[9] Rasmussen F, Mikkelsen D, Hancox RJ, et al. High-sensitive C-reactive protein is associated with reduced lung function in young adults. Eur Respir J. 2009;33(2):382–8. 19010993 10.1183/09031936.00040708

[10] Yoshii S, Tsuboi S, Morita I, et al. Temporal association of elevated C-reactive protein and periodontal disease in men. J Periodontol. 2009;80(5):734–9. 19405826 10.1902/jop.2009.080537

[11] Cullinan M, Ford P, Seymour G. Periodontal disease and systemic health: current status. Aust Dent J. 2009;54(s1):S62–9. 19737269 10.1111/j.1834-7819.2009.01144.x

[12] Zeng XT, Tu ML, Liu DY, et al. Periodontal disease and risk of chronic obstructive pulmonary disease: a meta-analysis of observational studies. PLoS ONE. 2012;7(10):e46508. 23094025 10.1371/journal.pone.0046508 PMC3477163

[13] Kucukcoskun M, Baser U, Oztekin G, et al. Initial periodontal treatment for prevention of chronic obstructive pulmonary disease exacerbations. J Periodontol. 2013;84(7):863–70. 23003917 10.1902/jop.2012.120399

[14] Zhou X, Han J, Liu Z, et al. Effects of periodontal treatment on lung function and exacerbation frequency in patients with chronic obstructive pulmonary disease and chronic periodontitis: a 2-year pilot randomized controlled trial. J Clin Periodontol. 2014;41(6):564–72. 24593836 10.1111/jcpe.12247

[15] Sundh J, Tanash H, Arian R, et al. Advanced dental cleaning is associated with reduced risk of COPD Exacerbations - A randomized controlled trial. Int J Chron Obstruct Pulmon Dis. 2021;16:3203–15. 34858021 10.2147/COPD.S327036 PMC8629912

[16] Apessos I, Voulgaris A, Agrafiotis M, et al. Effect of periodontal therapy on COPD outcomes: a systematic review. BMC Pulm Med. 2021;21(1):92. 33736634 10.1186/s12890-021-01429-2 PMC7976708

[17] ATS/ERS recommendations for standardized procedures for the. Online and offline measurement of exhaled lower respiratory nitric oxide and nasal nitric oxide, 2005. Am J Respir Crit Care Med. 2005;171(8):912–30. 15817806 10.1164/rccm.200406-710ST

[18] King GG, Bates J, Berger KI et al. Technical standards for respiratory oscillometry. Eur Respir J. 2020;55(2). 10.1183/13993003.00753-2019 31772002

[19] Miller MR, Hankinson J, Brusasco V, et al. Standardisation of spirometry. Eur Respir J. 2005;26(2):319–38. 16055882 10.1183/09031936.05.00034805

[20] Quanjer PH, Stanojevic S, Cole TJ, et al. Multi-ethnic reference values for spirometry for the 3-95-yr age range: the global lung function 2012 equations. Eur Respir J. 2012;40(6):1324–43. 22743675 10.1183/09031936.00080312 PMC3786581

[21] Ainamo J, Bay I. Problems and proposals for recording gingivitis and plaque. Int Dent J. 1975;25(4):229–35. 1058834

[22] O’Leary TJ, Drake RB, Naylor JE. The plaque control record. J Periodontol. 1972;43(1):38. 4500182 10.1902/jop.1972.43.1.38

[23] Tonetti MS, Greenwell H, Kornman KS. Staging and grading of periodontitis: framework and proposal of a new classification and case definition. J Periodontol. 2018;89(Suppl 1):S159–72. 29926952 10.1002/JPER.18-0006

[24] Sanz M, Herrera D, Kebschull M, et al. Treatment of stage I–III periodontitis—The EFP S3 level clinical practice guideline. J Clin Periodontol. 2020;47(S22):4–60. 32383274 10.1111/jcpe.13290 PMC7891343

[25] Preus HR, Gjermo P, Baelum V. A double-masked randomized clinical trial (RCT) comparing four periodontitis treatment strategies: 5-year clinical results. J Clin Periodontol. 2017;44(10):1029–38. 28796888 10.1111/jcpe.12793

[26] Molina A, Huck O, Herrera D, et al. The association between respiratory diseases and periodontitis: A systematic review and meta-analysis. J Clin Periodontol. 2023;50(6):842–87. 36606394 10.1111/jcpe.13767

[27] Kaminsky DA, Simpson SJ, Berger KI, et al. Clinical significance and applications of oscillometry. Eur Respiratory Rev. 2022;31(163):210208. 10.1183/16000617.0208-2021 PMC9488764 35140105

[28] Li L-Y, Yan T-S, Yang J, et al. Impulse oscillometry for detection of small airway dysfunction in subjects with chronic respiratory symptoms and preserved pulmonary function. Respir Res. 2021;22(1):68. 33627138 10.1186/s12931-021-01662-7 PMC7903610

[29] Gleeson K, Maxwell SL, Eggli DF. Quantitative aspiration during sleep in normal subjects. Chest. 1997;111(5):1266–72. 9149581 10.1378/chest.111.5.1266

[30] Hajishengallis G. Interconnection of periodontal disease and comorbidities: evidence, mechanisms, and implications. Periodontol 2000. 2022;89(1):9–18. 35244969 10.1111/prd.12430 PMC9018559

[31] Gomes-Filho IS, Passos JS, Seixas da Cruz S. Respiratory disease and the role of oral bacteria. J Oral Microbiol. 2010;2(1):5811. 10.3402/jom.v2i0.5811 PMC3084574 21523216

[32] Scannapieco FA. Role of oral bacteria in respiratory infection. J Periodontol. 1999;70(7):793–802. 10440642 10.1902/jop.1999.70.7.793

[33] Usher AKH, Stockley RA. The link between chronic periodontitis and COPD: a common role for the neutrophil? BMC Med. 2013;11(1):241. 24229090 10.1186/1741-7015-11-241 PMC4225606

[34] Tonetti MS, D’Aiuto F, Nibali L, et al. Treatment of periodontitis and endothelial function. N Engl J Med. 2007;356(9):911–20. 17329698 10.1056/NEJMoa063186

[35] Zhou QB, Xia WH, Ren J, et al. Effect of intensive periodontal therapy on blood pressure and endothelial microparticles in patients with prehypertension and periodontitis: a randomized controlled trial. J Periodontol. 2017;88(8):711–22. 28452620 10.1902/jop.2017.160447

[36] Quirynen M, De Soete M, Boschmans G, et al. Benefit of one-stage full-mouth disinfection is explained by disinfection and root planing within 24 hours: a randomized controlled trial. J Clin Periodontol. 2006;33(9):639–47. 16856902 10.1111/j.1600-051X.2006.00959.x

[37] Fonseca DC, Cortelli JR, Cortelli SC, et al. Clinical and microbiologic evaluation of scaling and root planing per quadrant and one-stage full‐mouth disinfection associated with Azithromycin or chlorhexidine: a clinical randomized controlled trial. J Periodontol. 2015;86(12):1340–51. 26252751 10.1902/jop.2015.150227

[38] Liu T, Chen YC, Jeng SL, et al. Short-term effects of chlorhexidine mouthwash and Listerine on oral Microbiome in hospitalized patients. Front Cell Infect Microbiol. 2023;13:1056534. 36816590 10.3389/fcimb.2023.1056534 PMC9932516

[39] Bescos R, Ashworth A, Cutler C, et al. Effects of chlorhexidine mouthwash on the oral Microbiome. Sci Rep. 2020;10(1):5254. 32210245 10.1038/s41598-020-61912-4 PMC7093448

[40] Tribble GD, Angelov N, Weltman R, et al. Frequency of tongue cleaning impacts the human tongue Microbiome composition and enterosalivary circulation of nitrate. Front Cell Infect Microbiol. 2019;9:39. 30881924 10.3389/fcimb.2019.00039 PMC6406172

[41] Qvarnström B, Engström G, Frantz S et al. Impulse oscillometry indices in relation to respiratory symptoms and spirometry in the Swedish cardiopulmonary bioimage study (SCAPIS). ERJ Open Res. 2023:00736–2022. 10.1183/23120541.00736-2022 PMC10518858 37753278

[42] Eisner MD, Anthonisen N, Coultas D, et al. An official American thoracic society public policy statement: novel risk factors and the global burden of chronic obstructive pulmonary disease. Am J Respir Crit Care Med. 2010;182(5):693–718. 20802169 10.1164/rccm.200811-1757ST

[43] Hyman JJ, Reid BC. Cigarette smoking, periodontal disease, and chronic obstructive pulmonary disease. J Periodontol. 2004;75(1):9–15. 15025211 10.1902/jop.2004.75.1.9

[44] Abdo M, Kirsten A-M, von Mutius E et al. Minimal clinically important difference for impulse oscillometry in adults with asthma. Eur Respir J. 2023:2201793. 10.1183/13993003.01793-2022 PMC10160799 36758985

