# Supplementary material for: Effect of periodontal therapy on lung function: a twelve-month follow-up intervention study
Source: Respir Res. 2025 May 3;26:172. doi: 10.1186/s12931-025-03246-1 (PMC12049769; doi:10.1186/s12931-025-03246-1)
Supplement: Supplementary file 1 — Supplementary Material 1 [file 12931_2025_3246_MOESM1_ESM.docx]

**Supplementary Appendix: Effect of periodontal therapy on lung function: A twelve-month follow-up intervention study**

Anders Røsland^1,2^, Randi J. Bertelsen^3,4^, Joachim Heinrich^5^, Stein Atle Lie^2^, Andrei Malinovschi^6^, Dagmar F Bunæs^1,2^

^1^University Of Bergen, Department of Clinical Dentistry, Section of Periodontics, Bergen, Norway. ^2^University of Bergen, Department of Clinical Dentistry, Centre for Translational Oral Research (TOR), Bergen, Norway. ^3^University Of Bergen, Department of Clinical Science, Bergen, Norway. ^4^Oral health centre of expertise in Western Norway, Bergen, Norway. ^5^University of Munich (LMU), Institute of Occupational, Social, and Environmental Medicine, Munich, Germany, ^6^Uppsala University, Department of Medical Sciences, Clinical Physiology, Uppsala, Sweden.

Contents

[**Methods** 2](#_Toc195275381)

[**Results** 2](#_Toc195275382)

[**Table S1** 2](#_Toc195275383)

[**Figure S1** 4](#_Toc195275384)

[**Figure S2** 5](#_Toc195275385)

[**Figure S3** 6](#_Toc195275386)

[**Figure S4** 7](#_Toc195275387)

[**Figure S5** 8](#_Toc195275388)

## **Methods**

**Pre study tests**

One intra-calibrated dentist performed all oral examinations and interventions (AR). Intra-examiner reproducibility was validated by registration of periodontal pocket depths (PD) and clinical attachment loss (CAL), at two time-points, six sites per tooth in a sample of eight patients with 1344 sites in total. Intra-class correlation coefficients for the repeated measures for PD and CAL were 0.98 and 0.91, respectively.

**Lung function and FeNO measurements**

FOT measurements: A minimum of three valid measurements was required for a valid patient test, but no more than eight repetitions were performed. For a test to be acceptable, optimally there has to be <10% difference in R_5_ between all manoeuvres according to the same guidelines, but in a recent study it was suggested that measurements with differences up to <15%-20% could be usable (Harkness, L. M. *et al.* Within-session variability as quality control for oscillometry in health and disease. *ERJ Open Research* **7**, 00074-02021, doi:10.1183/23120541.00074-2021 (2021)).

Participants were seated in an upright position, wearing a nose clip, and asked to place their hands around their cheeks. FOT data were collected for 30-60 sec during tidal breathing. A single measurement of FeNO levels (ppb) were collected using NIOX VERO^TM^ (Circassia, Oxford, UK). The participants were instructed to refrain from eating, drinking, and exercising one hour prior to the examination.

**Other patient related assessment**

Height (cm) and weight (kg) were measured, and body mass index (BMI) was calculated as weight/height^2^ (kg/m^2^). A questionnaire was used to record socioeconomic status, self-perceived oral and respiratory health, physical activity, and use of antibiotics. For questionnaire distribution and data collection, the web-based platform EasyTrial.net (EasyTrial ApS, Denmark) was used. Two tubes of blood serum (2 x 8.5 ml) were collected during the examination at Research Unit for Health Surveys, University of Bergen.

**Periodontal therapy**

*Oral hygiene kit for home use*

To support optimal oral hygiene, participants were provided with an oral hygiene kit comprising toothbrushes, interdental brushes, solo brushes, and dental floss (Curaprox, Curaden, Switzerland, Johnson&Johnson, USA). Additionally, they received toothpaste (Curaprox enzycal 1450, Curaden, Switzerland) and plaque disclosing tablets for self-assessment of oral hygiene (Curaprox, Curaden, Switzerland).

## **Results**

**Table S1. Baseline characteristics stratified by participants who dropped out of the study versus those who completed the trial (from T0 to T3).**

| Variables | Non-dropouts | Drop-outs | *p-value* |
| --- | --- | --- | --- |
| Participants | 48 (77.4%) | 14 (22.6%) |  |
| Mean age (SD) | 36.5 (5.9) | 33.3 (6.9) | 0.09 |
| Sex  Female | 28 (58.3%) | 8 (57.1%) | 0.94 |
| Education at University Level | 41 (87.2%%) | 10 (77.0%) | 0.30 |
| Mean body mass index in kg/m^2^ (SD) | 26.6 (4.82) | 29.9 (5.8) | 0.032 |
| Mean height in cm (SD) | 172.5 (10.2) | 170.5 (8.8) | 0.50 |
| Mean weight in kg (SD) | 79.7 (17.9) | 87.7 (20.3) | 0.16 |
| Absolute values of spirometry variables  Mean FEV_1_ (SD)  Mean FVC (SD)  Mean FEV_1_/FVC (SD)  %predicted spirometry variables  Mean %predicted FEV_1_ (SD)  Mean %predicted FVC (SD) | 3.57 (0.77)  4.58 (1.05)  0.78 (0.05)  94.85 (11.14)  99.41 (12.02) | 3.45 (0.77)  4.33 (0.90)  0.79 (0.05)  92.40 (9.74)  95.90 (8.51) | 0.62  0.41  0.40  0.45  0.30 |
| Absolute values of oscillometry variables  Mean R_5_ (SD)  Mean R_11_ (SD)  Mean R_19_ (SD)  %predicted oscillometry variables  Mean R_5_ %predicted (SD)  Mean R_11_ %predicted (SD)  Mean R_19_ %predicted (SD) | 3.20 (1.17)  2.98 (0.91)  2.79 (0.76)  105.1 (27.8)  104.4 (24.7)  94.6 (20.3) | 3.35 (0.74)  3.10 (0.57)  2.73 (0.56)  93.4 (17.2)  93.7 (14.9)  86.8 (14.0) | 0.60  0.73  0.78  0.18  0.17  0.23 |
| Fraction of Exhaled Nitric Oxide (FeNO)  Mean FeNO in parts per billion (SD) | 18.5 (13.7) | 20.8 (16.9) | 0.60 |
| Severity of periodontitis  Stage I  Stage II | 9 (18.7%)  39 (81.3%) | 4 (28.6%)  10 (71.4%) | 0.42 |
| Periodontal variables  Mean BoP in % (SD)  Mean PI in % (SD)  Mean PD in mm (SD)  Mean CAL in mm (SD) | 63.5 (14.1)  62.1 (10.2)  2.51 (0.18)  2.75 (0.17) | 61.7 (13.1)  61.8 (10.6)  2.36 (0.18)  2.55 (0.17) | 0.70  0.92  0.01  <0.001 |
| Mean high-sensitive C-reactive protein (mg/L) | 1.50 (2.14) | 2.65 (3.19) | 0.20 |

Table S1. Data are presented as *n* (%) or mean and standard deviations (SD). FEV_1_/FVC is unitless and has the formula FEV_1_(L)/FVC (L). Percent predicted values were calculated from the 2012 Global Lung function Initiative P-values are calculated by t-test (continuous variables) and Chi-square tests (categorical variables). Abbreviations: FEV_1_: forced expiratory volume in the first second, FVC: forced vital capacity, R_5_: airway resistance measured at 5Hz, R_11_: airway resistance measured at 11Hz, R_19_: airway resistance measured at 19Hz. BoP: bleeding on probing, PI: plaque index, PD: periodontal pocket depth, CAL: clinical attachment loss

**Figure S1. Change in oral parameters from T0 to T3 stratified by sex**


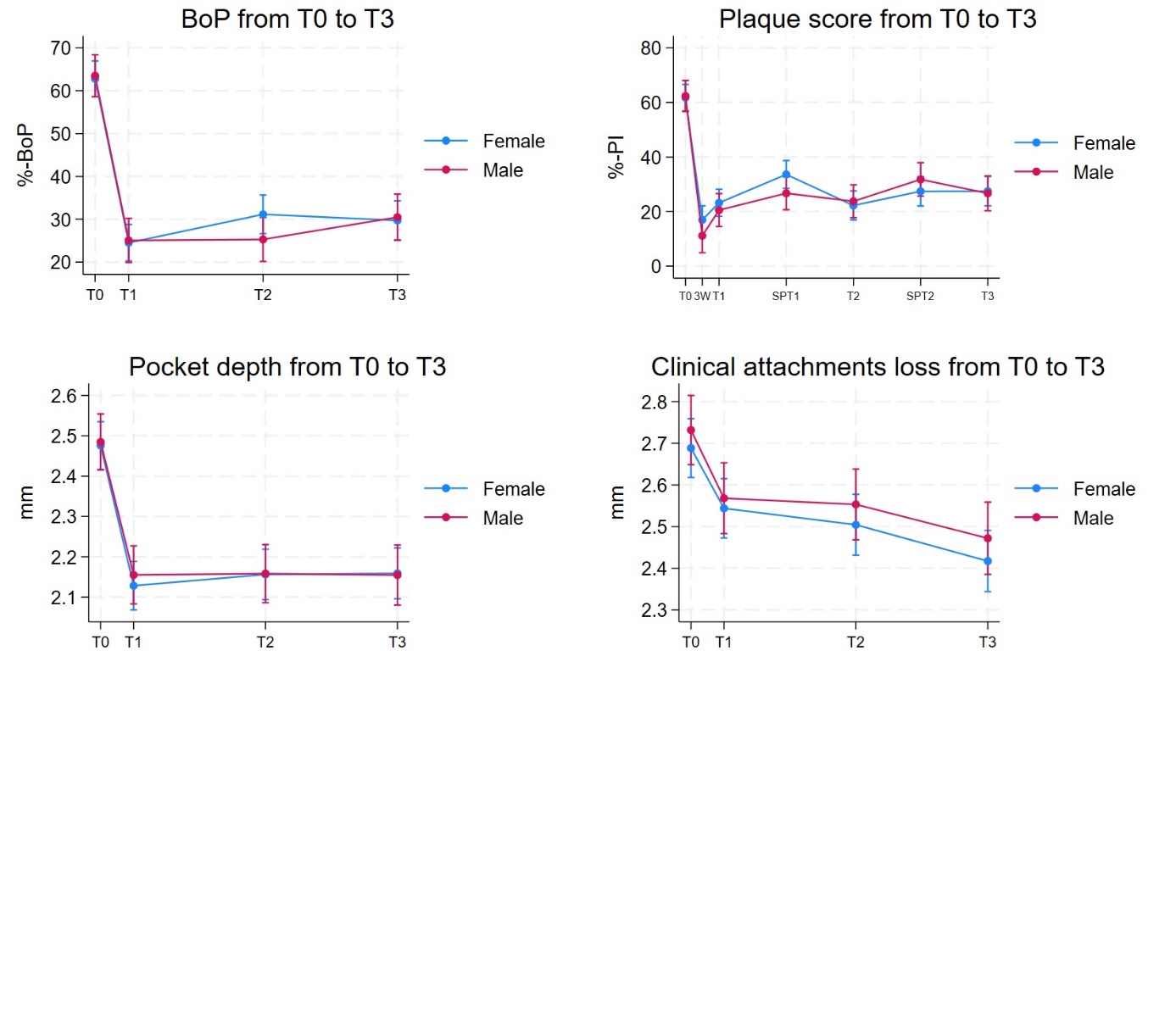


Figure S1 illustrates changes in oral parameters from T0 to T3, stratified by sex. Graphs showing marginal means with 95% confidence intervals. The interaction term between sex and time was not statistically significant for any of the parameters; BoP (bleeding on probing), plaque score, pocket depth, or clinical attachment level (all p-values>0.05).

**
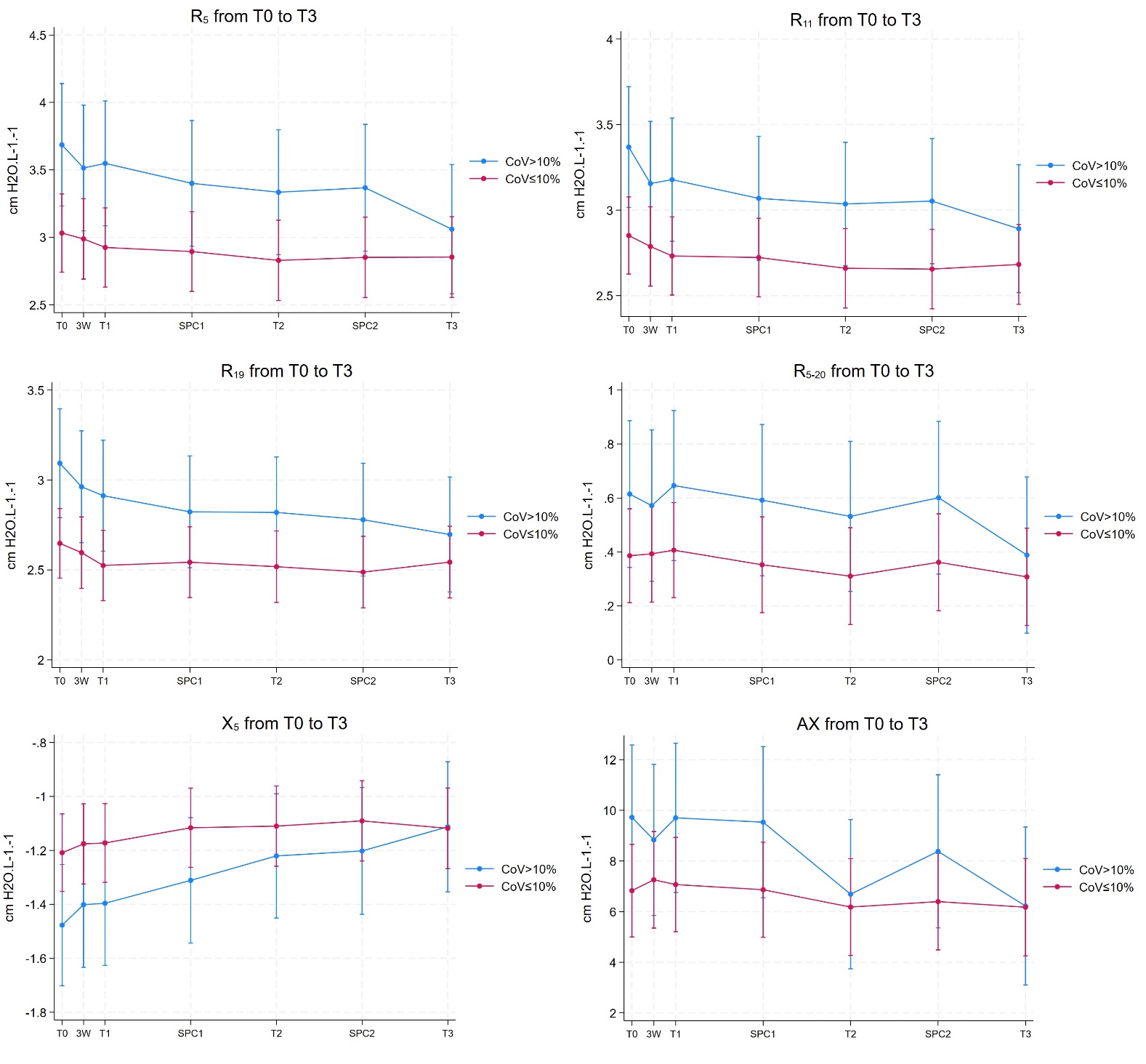
****Figure S2. Changes in FOT variables from T0 to T3, stratified by patients with a baseline coefficient of variance (CoV) at R5>10% and those with a CoV<10%.**

Figure S2 illustrates changes FOT parameters from T0 to T3, stratified by patients with a baseline coefficient of variance (CoV) at R5<10% (red line) and those with a CoV>10% (blue line). Graphs showing marginal means with 95% confidence intervals. The interaction term between the two groups and time was not statistically significant for any of the parameters (all p-values>0.05). Results from mixed effects model.

**
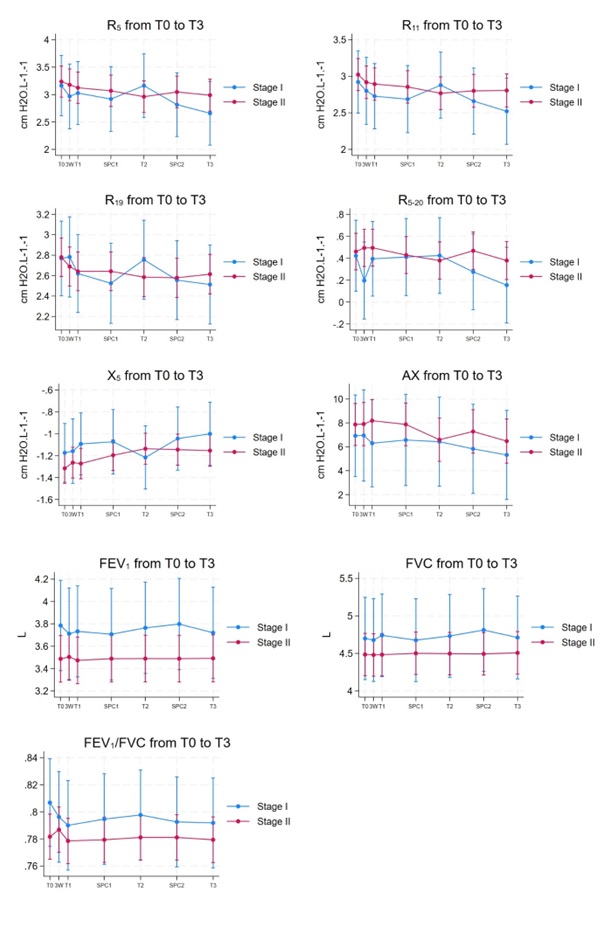
****Figure S3. Changes in FOT- and spirometry variables from T0 to T3, stratified by periodontal diagnose.**

Figure S3 illustrates changes in oral FOT- and spirometry parameters from T0 to T3, stratified by periodontal diagnose. Graphs showing marginal means with 95% confidence intervals. Results from mixed effects model. The interaction term between periodontal diagnose and time was not statistically significant for any of the parameters (all p-values>0.05).

**Figure S4. Change in CRP from T0 to T3**


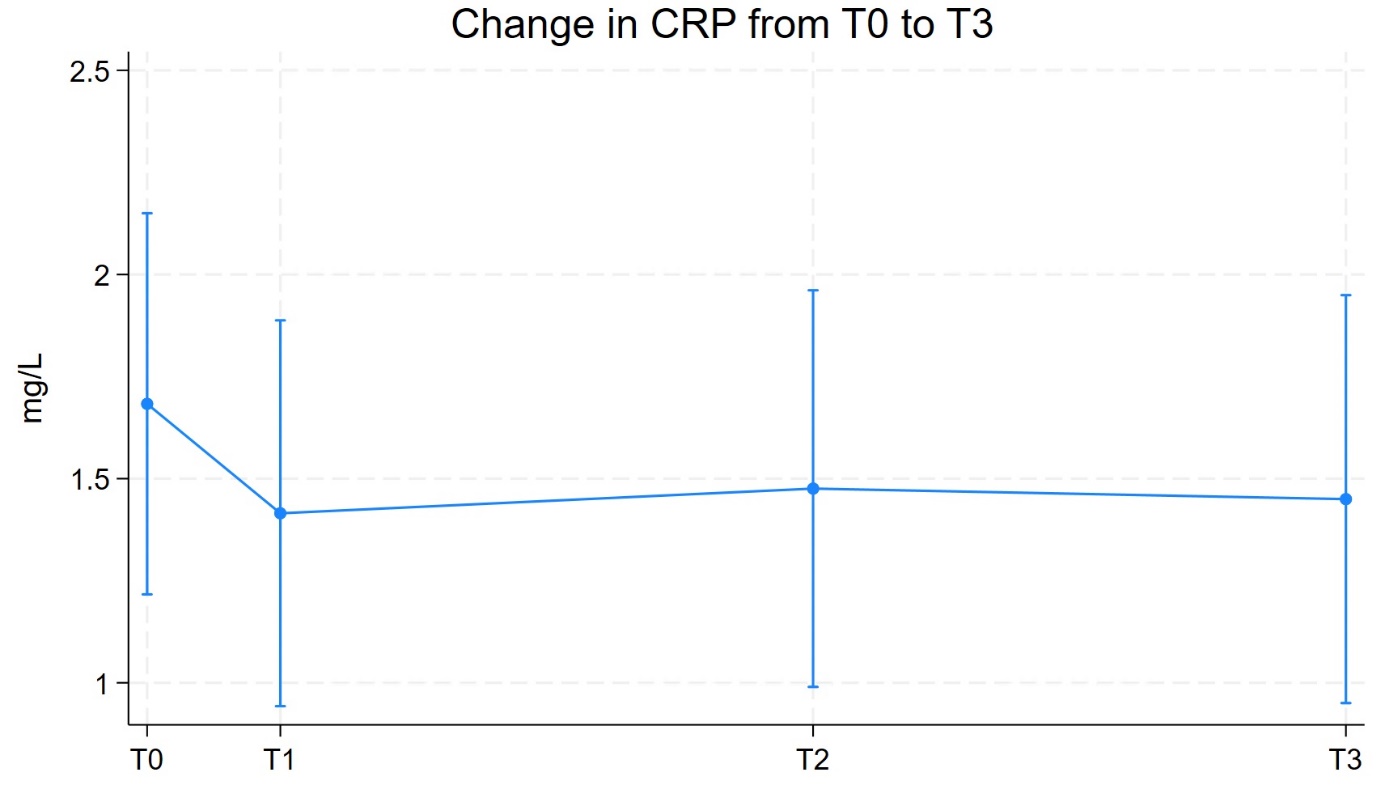


Figure S4 illustrates the changes in C-reactive protein (CRP) levels (mg/L) from T0 to T3. Graph showing marginal means with 95% confidence intervals. Results from mixed effects model (p>0.05).

**
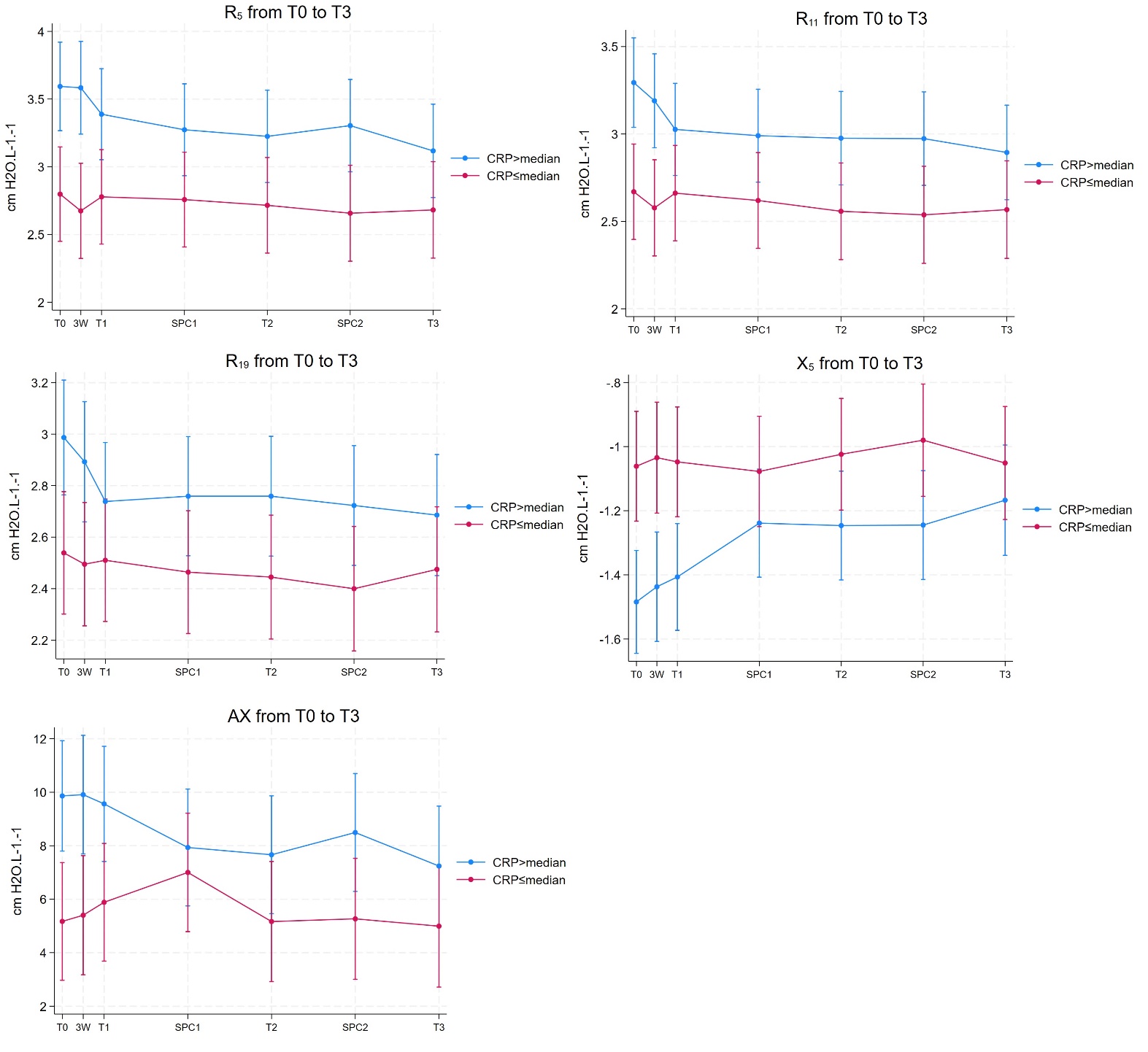
****Figure S5. Changes in oscillometry variables from T0 to T3, stratified by patients with a baseline CRP>median and those with a baseline CRP<median.**

Figure S5 illustrates the changes in C-reactive protein (CRP) (mg/L) from T0 to T3 s stratified by patients with a baseline CRP>median (blue line) and those with a baseline CRP<median (red line). Graphs showing marginal means with 95% confidence intervals. The interaction term between the two groups and time was statistically significant for R_5_ (p=0.04), X_5_ (p=0.002) and AX (p=0.04). Results from mixed effects model.
